# Cigarette smoking and tooth loss experience among young adults: a national record linkage study

**DOI:** 10.1186/1471-2458-7-313

**Published:** 2007-11-02

**Authors:** Miki Ojima, Takashi Hanioka, Keiko Tanaka, Hitoshi Aoyama

**Affiliations:** 1 Department of Preventive Dentistry, Graduate School of Dentistry, Osaka University, Osaka, Japan; 2 Department of Preventive and Public Health Dentistry, Fukuoka Dental College, Fukuoka, Japan; 3 Department of Public Health, Faculty of Medicine, Fukuoka University, Fukuoka, Japan; 4 Tochigi Prefectural Medical and Social Welfare College, Utsunomiya, Tochigi, Japan

## Abstract

**Background:**

Various factors affect tooth loss in older age including cigarette smoking; however, evidence regarding the association between smoking and tooth loss during young adulthood is limited. The present study examined the association between cigarette smoking and tooth loss experience among adults aged 20–39 years using linked data from two national databases in Japan.

**Methods:**

Two databases of the National Nutrition Survey (NNS) and the Survey of Dental Diseases (SDD), which were conducted in 1999, were obtained from the Ministry of Health, Labor and Welfare with permission for analytical use. In the NNS, participants received physical examinations and were interviewed regarding dietary intake and health practices including cigarette smoking, whereas in the SDD, participants were asked about their frequency of daily brushing, and received oral examinations by certified dentists. Among 6,805 records electronically linked via household identification code, 1314 records of individuals aged 20 to 39 years were analyzed. The prevalence of 1+ tooth loss was compared among non-, former, and current smokers. Multiple logistic regression models were constructed including confounders: frequency of tooth brushing, body mass index, alcohol consumption, and intake of vitamins C and E.

**Results:**

Smoking rates differed greatly in men (53.3%) and women (15.5%). The overall prevalence of tooth loss was 31.4% (31.8% men and 31.1% women). Tooth loss occurred more frequently among current smokers (40.6%) than former (23.1%) and non-smokers (27.9%). Current smoking showed a significant association with 1+ tooth loss in men (adjusted OR = 2.21 [1.40–3.50], P = 0.0007) and women (1.70 [1.13–2.55], P = 0.0111). A significant positive exposure-related relationship between cigarette smoking status and tooth loss was observed (P for trend < 0.0001 and 0.0004 in men and women, respectively). Current smoking was also associated with the prevalence of decayed teeth (1.67 [1.28–2.20], P = 0.0002).

**Conclusion:**

An association between cigarette smoking and tooth loss was evident among young adults throughout Japan. Due to limitations of the available variables in the present databases, further studies including caries experience and its confounders should be conducted to examine whether smoking is a true risk of premature tooth loss in young adults.

## Background

The loss of many teeth often reduces the quality of life; embarrassment and self-consciousness limit social interaction and communication [1]. Because of chewing problems and decreased masticatory function, a limitation in food selection may occur, resulting in nutritionally poor diets. Poor nutrition might contribute to an increased risk of several systemic diseases such as cardiovascular diseases [2] and hypertension [3]. Tooth loss may be a significant problem related to general health and the quality of life.

Many studies demonstrated that cigarette smokers have more missing teeth and experience greater rates of tooth loss than non-smokers. Most of these studies regarding the association between smoking and tooth loss concentrated on middle aged and elderly populations and, among them, several studies observed nationally represented population [4-6] and longitudinal cohort [7]. On the other hand, the effect of smoking in young adults would be more apparent than that in older adults because the effects of confounding factors, which may be age-dependent, would be less apparent.

Smoking is a global problem. In several countries, the association between smoking and tooth loss in young adults has been examined [8-10]; however, the sample population was limited to specific age groups, gender and region. Health consequences of smoking at the national level should be evaluated using nationally representative samples of the country.

Data on cigarette smoking and dental status have been accumulated in two independent national surveys in Japan, and record linkage using a common household number in the two surveys is possible. The aim of the present study was to examine the association between cigarette smoking and tooth loss experience among adults aged 20–39 years using linked data from the two national databases.

## Methods

Databases of the National Nutrition Survey (NNS) and the Survey of Dental Diseases (SDD) from 1999 were obtained from the Ministry of Health, Labor and Welfare with permission for analytical use. The SDD, which involved the same sample population as that of the NNS, is conducted every six years. Data were collected from persons aged 1 year or older from approximately 5000 households in 300 districts. The background and general procedure of the surveys were described previously [11].

Smoking status was defined in the questionnaires of the NNS as: "current smoker", an individual who currently smokes cigarettes daily or occasionally; "former smoker", an individual who has smoked cigarettes at some point in their life, but who currently does not smoke; "non-smoker", an individual who was an experimental smoker or who has never smoked cigarettes. Current smokers were asked about cigarette consumption in terms of the number of cigarettes per day and duration of smoking.

In the SDD, calibrated dentists examined and recorded the status of each tooth which was present in the oral cavity regardless of the degree of eruption. Lost teeth were defined as a permanent tooth (excluding third molars) lost due to extraction or dropout. Though reasons for extraction were not surveyed, teeth lost through extraction due to orthodontic treatment (e.g., bilateral missing of first premolars) were not included. Decayed and filled teeth were diagnosed with a mirror and a Community Periodontal Index probe in the absence of an air jet. Initial and manifest caries lesions for all teeth were recorded. Secondary caries was categorized into decayed teeth. Coronal and root caries were not differentiated.

We selected several variables *a priori *as possible confounders from the two databases in order to control for the effect of behavioral and lifestyle factors on tooth loss. The frequency of daily brushing was divided into two groups: more than twice and less than twice. The body mass index (BMI) was calculated as an indicator of obesity. BMI was divided into two groups: <25.0 and ≥25.0. Alcohol consumption was classified into three groups: current, former, and never. Based on the Recommended Dietary Allowances for Japanese (6^th ^Revision), vitamin C and vitamin E intakes were categorized into two groups: deficiency and sufficiency. The reference value of vitamin C was 100 mg/day, and those of vitamin E for males and females were 10 mg/day and 8 mg/day, respectively.

Records in the SDD database were electronically linked to those in the NNS database using the household identification number as the linking variable. All links were checked via comparison of the information on birth month and sex between the two electronic databases. In the event of a mismatch of these variables, such cases were evaluated by a manual search of records among the same household. Among records of 6903 subjects in the SDD and of 12,763 subjects in the NNS, 6805 records were linked successfully. Analyses were limited to 1314 subjects aged 20 to 39 years without missing values.

The prevalence of 1+ tooth loss was compared among non-, former, and current smokers on the basis of gender and study variables. Bivariate analyses were conducted to determine associations between selected variables as well as smoking and the prevalence of tooth loss using the chi-square test. The multiple logistic regression models were constructed, stratified by sex, including frequency of daily brushing, BMI, current smoking, vitamin C and E intakes, and alcohol consumption as confounding factors. Adjusted odds ratios (ORs) for tooth loss for each variable were calculated. The dose-response relationship was evaluated using life-time exposure as the Brinkman Index, which was calculated by daily consumption times years of smoking. Former smokers were excluded from this analysis because data on the number of years after cessation of smoking were not available. Trend analysis was performed by entering categories of life-time exposure as a continuous variable. SPSS for Windows (SPSS Inc., Chicago) was utilized.

Since caries is the predominant reason for extraction in the study population [12], the association between smoking status and caries prevalence was further examined to investigate the hypothesis that caries experience may modulate subsequent tooth loss possibly due to smoking.

## Results

### Distribution of smokers by study variables

Table 1 shows the prevalence of current, former, and non-smokers by study variables among adults aged 20–39 years. The overall percentage of current smokers was 29.6% of the study population (55.3% males and 15.5% females). Current smokers were relatively predominant in the following groups in comparison with counterpart groups: daily brushing less than twice (142/286; 49.7% vs 247/1028; 24.0%), BMI ≥ 25.0 (101/245; 41.2% vs 288/1069; 26.9%) and current drinking (169/288; 58.7% vs 203/992; 20.5%).

**Table 1:** Number of subjects according to smoking status by study variables

		Smoking status		
		
Variables	Total	Non-	Former	Current
Gender				
males	465	170	38	257
females	849	677	40	132
Frequency of daily brushing				
more than twice	1028	718	63	247
less than twice	286	129	15	142
BMI				
< 25.0	1069	717	64	288
≥25.0	245	130	14	101
Alcohol consumption				
never	992	744	45	203
former	34	10	7	17
current	288	93	26	169
Vitamin C intake				
≥100 mg	596	403	33	160
< 100 mg	718	444	45	229
Vitamin E intake*				
≥10 or 8 mg	671	451	37	183
< 10 or 8 mg	643	396	41	206

All	1314	847	78	389

*10 mg for males and 8 mg for females

### Bivariate associations between tooth loss and decayed teeth, and study variables

The prevalence of tooth loss and decayed teeth was compared using the study variables in Table 2. Smoking and each variable, with the exception of gender and vitamin intake, were associated with the prevalence of tooth loss (P < 0.05). All variables including smoking were associated with the prevalence of decayed teeth (P < 0.05). Subjects who had lost teeth were more frequently those with decayed teeth than those without. Also, subjects with decayed teeth were more likely to be those with lost teeth than those without.

**Table 2:** Results from bivariate analyses of tooth loss and decayed teeth, and study variables

Study variables	tooth loss	decayed teeth
	
	p-value^†^	p-value^‡^
Smoking status (non vs. former or current)	0.0002	< 0.0001
Gender (females vs males)	0.7843	< 0.0001
Frequency of brushing (≥ 2 times vs. < 2 times)	0.0390	0.0001
Body mass index (< 25.0 vs. ≥ 25.0)	0.0012	< 0.0001
Alcohol consumption (never vs. former or current)	0.0084	< 0.0001
Intake of vitamin C (≥ 100 mg vs. < 100 mg)	0.5403	0.0037
Intake of vitamin E*(≥ 10/8 mg vs. < 10/8 mg)	0.4472	0.0273
		
Tooth loss (1+ vs. 0)	-	< 0.0001
Decayed teeth (1+ vs. 0)	< 0.0001	-

*10 mg for males and 8 mg for females

† differences in prevalence of 1+ tooth loss

‡ differences in prevalence of 1+ decayed teeth

### Prevalence of tooth loss by smoking status

Table 3 shows the prevalence of 1+ tooth loss by smoking status among adults aged 20–39 years. Overall, 31.4% of subjects had lost one or more teeth, and the mean tooth loss was 0.6 ± 1.4 teeth. The distribution of subjects with 1–3 lost teeth, 4–6 and ≥7 was 27.2%, 3.2% and 1.0%, respectively. The prevalence of tooth loss was similar in males (31.8%) and females (31.1%). Tooth loss occurred more frequently among current smokers (40.6%) than former (23.1%) and non-smokers (27.9%) in the overall population. A significant difference was observed among smoking status groups (p < 0.0001). In both genders, significant differences were detected among smoking status groups (p = 0.0005 and 0.0022, in males and females, respectively).

**Table 3:** Comparison of prevalence (%) of 1+ tooth loss by smoking status

		Smoking status			
			
	Total	Non-	Former	Current	P value
Males	31.8 (148/465)	21.8 (37/170)	26.3 (10/38)	39.3 (101/257)	0.0005
Females	31.1 (264/849)	29.4 (199/677)	20.0 (8/40)	43.2 (57/132)	0.0022

Overall	31.4 (412/1314)	27.9 (236/847)	23.1 (18/78)	40.6 (158/389)	<0.0001

### Association between cigarette smoking and prevalence of tooth loss

Adjusted ORs for the prevalence of tooth loss are presented in Table 4. Current smoking was significantly correlated with tooth loss in males (OR = 2.21, 95% CI; 1.40–3.50) and females (OR = 1.70, 95% CI; 1.13–2.55), following consideration of possible confounding factors. However, the association with tooth loss was not significant with respect to former smoking. BMI and the frequency of daily brushing were significantly associated with the prevalence of 1+ tooth loss in females.

**Table 4:** Adjusted odds ratio (OR) and 95% confidence interval (CI) for prevalence of tooth loss

		Males		Females	
		
Study variable	Criteria	Adjusted OR (95% CI)	P value	Adjusted OR (95% CI)	P value
Smoking status	Non-	1.00 (Reference)		1.00 (Reference)	
	Former	1.25^† ^(0.55–2.86)	0.5971	0.52^† ^(0.23–1.18)	0.1197
	Current	2.21^† ^(1.40–3.50)	0.0007	1.70^† ^(1.13–2.55)	0.0111
Frequency of brushing	≥2 times	1.00 (Reference)		1.00 (Reference)	
	< 2 times	0.97 (0.64–1.48)	0.8945	1.59 (1.03–2.44)	0.0350
Body mass index	< 25.0	1.00 (Reference)		1.00 (Reference)	
	≥25.0	1.28 (0.83–1.96)	0.2640	2.00 (1.31–3.04)	0.0013
Alcohol consumption	Never	1.00 (Reference)		1.00 (Reference)	
	Former	1.05 (0.34–3.30)	0.9287	1.95 (0.75–5.06)	0.1685
	Current	1.17 (0.77–1.78)	0.4565	1.48 (0.90–2.44)	0.1205
Intake of Vitamin C	≥100 mg	1.00 (Reference)		1.00 (Reference)	
	< 100 mg	1.01 (0.67–1.53)	0.9559	0.81 (0.59–1.11)	0.1899
Intake of Vitamin E*	≥10/8 mg	1.00 (Reference)		1.00 (Reference)	
	< 10/8 mg	1.16 (0.77–1.76)	0.4756	1.10 (0.81–1.50)	0.5398

*10 mg for males and 8 mg for females

^†^Adjusting for frequency of brushing, body mass index, alcohol consumption, intake of Vitamin C and E

### Exposure-related relationship between cigarette smoking and prevalence of tooth loss

An exposure-related relationship was demonstrated among non- and current smokers, stratified by gender in Table 5. The prevalence of 1+ tooth loss and adjusted OR increased with lifetime exposure in both genders. Current smoking with a Brinkman Index of ≥200 was significantly associated with an increased prevalence of 1+ tooth loss. A significant positive exposure-related relationship between cigarette smoking status and tooth loss was observed (P for trend < 0.0001 and 0.0004 in males and females, respectively).

**Table 5:** Adjusted odds ratio (OR) and 95% confidence interval (CI) tooth loss by lifetime exposure to smoking

	Lifetime exposure*	Prevalence of 1 + tooth loss (%)	Adjusted OR^† ^(95%CI)	P value
Males	0	21.8 (37/170)	1.00 (Reference)	
	1–199	26.9 (29/108)	1.34 (0.76–2.36)	0.3134
	200–399	42.7 (41/96)	2.75 (1.57–4.83)	0.0004
	400+	58.5 (31/53)	5.17 (2.59–10.3)	<0.0001
	
	P for trend			<0.0001

Females	0	29.4 (199/677)	1.00 (Reference)	
	1–199	36.2 (38/105)	1.31 (0.84–2.06)	0.2379
	200+	70.4 (19/27)	5.34 (2.24–12.7)	0.0002
	
	P for trend			0.0004

*Brinkman Index

^†^Adjusting for frequency of brushing, body mass index, alcohol consumption, intake of Vitamin C and E

### Association between cigarette smoking and prevalence of decayed teeth

Based on the finding that the prevalence of 1+ tooth loss between current and non-smokers clearly differed in adults aged 20–39 years, the following analyses were further performed in terms of caries, because caries is a major reason for tooth extraction in the age group. Only decayed teeth were considered as the dependent variable due to the high prevalence of filled teeth (96.7%). Prevalence and adjusted ORs and 95% CIs for decayed teeth among adults aged 20–39 years are presented in Table 6. The association between smoking and decayed teeth was significant in both genders. Adjusted ORs were 1.87(95% CI; 1.24–2.84), 1.56(95% CI; 1.04–2.33), and 1.67(95% CI; 1.28–2.20), in males, females, and overall, respectively.

**Table 6:** Prevalence and adjusted OR of decayed teeth

	Smoking status	Prevalence (%) of decayed teeth	Adjusted OR^† ^(95%CI)	P value
Males	Non-	37.1 (63/170)	1.00 (Reference)	
	Former	44.7 (17/38)	1.34 (0.64–2.80)	0.4362
	Current	55.6 (143/257)	1.87 (1.24–2.84)	0.0030
Females	Non-	33.1 (224/677)	1.00 (Reference)	
	Former	40.0 (16/40)	1.10 (0.56–2.19)	0.7780
	Current	46.2 (61/132)	1.56 (1.04–2.33)	0.0308
Overall	Non-	33.9 (287/847)	1.00 (Reference)	
	Former	42.3 (33/78)	1.25 (0.77–2.04)	0.3677
	Current	52.4 (204/389)	1.67 (1.28–2.20)	0.0002

^†^Adjusting for frequency of brushing, body mass index, alcohol consumption, intake of Vitamin C and E

## Discussion

In the present study, a significant association between current smoking and tooth loss prevalence was demonstrated among young adults aged 20–39 years in Japan. Following adjustment for confounding factors, the odds of 1+ tooth loss was approximately 2 times greater in current smokers than non-smokers. Furthermore, a dose-response relationship with lifetime exposure to cigarette smoking was evident. Smokers are likely to show unhealthy behavior and a negative attitude towards general health [13]. In the present study, after adjusting for behavioral and lifestyle factors, the relation of smoking to tooth loss was evident, consistent with the previous study [8]. Vitamin C and E intake was not independently associated with tooth loss. The effect of vitamin C and E intake on tooth loss, if any, may be observed via periodontitis [14,15]. Caries is the predominant reason for extraction in the study population [12]. The design of the present study was cross-sectional. The time sequence between smoking status and tooth loss could not be assessed because the point in time when the teeth were lost was not available; therefore, a causal association between smoking and tooth loss should not be assumed.

Few studies have suggested an association between smoking and tooth loss in young adults [8-10]; however, the population was not a nationally representative sample and the analysis was based on self-reported tooth loss. In the present study, 1314 samples throughout Japan were analyzed, and the number of teeth was assessed by clinical examination. The 1314 records consisted of 42% of those subjects aged 20–39 years in the NNS. Participants of the dental examination may be limited and biased. However, this sample was similar to the NNS sample with respect to smoking prevalence by sex and age (data not shown). Several confounders were not controlled in the multivariate models. Socio-economic status (SES) and dental service use were not included in the surveys; consequently, this parameter was not considered in the present investigation. In a cohort study of young New Zealanders, SES inequalities in tooth loss appear to begin early in the life course, and are modified by individuals' SES and dental service use [16]. Few studies have examined the relation between socioeconomic factors and smoking in the Japanese population. Association of socioeconomic status with smoking was reported in the recent article [17]; however, the effect of socioeconomic disadvantage on smoking was controversial and much less than in other industrialized counties in the 1990s [18-20]. No significant difference was found in smoking prevalence by SES and the frequency of dental check-ups among Japanese in their twenties in 1997 [21]. The effect of these factors may have been weak at the time when the surveys were conducted, and in the study population.

A national survey in Japan reported that about 85% of permanent tooth extractions were due to dental caries and its sequela, and periodontal disease [12]. Caries-associated tooth loss increased in the late twenties and early thirties in a birth cohort study of New Zealanders [22]. In the study population, current smokers may loose teeth via extraction due to dental caries rather than periodontal disease. Actually, no significant difference was observed in the prevalence of periodontitis between current and non- smokers in the same population [11]. Several hypothesized mechanisms underlie the relationship between active smoking and dental caries [23], e.g., impairment of the immune system, alteration of the bacterial profile, and salivary function. These observations may be associated with an increase of endodontic diseases [24], which is also a reason for extraction in young adulthood.

The association between active smoking and dental caries is controversial [23]. Methodological considerations limit interpretation of the findings of the aforementioned investigations. In particular, many studies did not control for potential confounding factors of these associations, such as less effective dental hygiene and plaque removal [25]. We found that the independent association between cigarette smoking and the prevalence of decayed teeth following adjustment for behavioral factors including oral hygiene practices. Biological and prospective epidemiological studies are further required for assessments of the effect of smoking on carious lesion formation.

## Conclusion

The association between cigarette smoking and tooth loss was evident among young adults throughout Japan. Due to limitations of the available variables in the present databases, further studies should be conducted to examine whether smoking is a true risk of premature tooth loss in young adults by employing variables of caries experience and its confounders.

## Competing interests

The author(s) declare that they have no competing interests.

## Authors' contributions

MO performed the statistical analysis and drafted the manuscript. TH conceived of the study, and participated in its design and coordination and helped to draft the manuscript. KT helped to draft the manuscript and analyze the data. HA participated in the design and coordination of the study. All authors read and approved the final manuscript.

## Pre-publication history

The pre-publication history for this paper can be accessed here:


